# αβ-Double Negative CD4/CD8 (CD56) T cell (DNTs) in metastatic melanoma: basal frequency and behaviour during Ipilimumab treatment. Preliminary evaluations

**DOI:** 10.1186/1479-5876-13-S1-O10

**Published:** 2015-01-15

**Authors:** Giacoma De Tullio, Sabino Strippoli, Rosa Angarano, Vincenza De Fazio, Nicola Sgherza, Antonio Negri, Anna Albano, Pasquale Iacopino, Attilio Guarini, Michele Guida

**Affiliations:** 1 Hematology Unit, National Cancer Research Centre,Istituto Tumori “Giovanni Paolo II”,Bari – Italy; 2 Medical Oncology Department National Cancer Research Centre, Istituto Tumori “Giovanni Paolo II”, Bari – Italy; 3 Clinical Institute “Prof. R. De Blasi”, Reggio Calabria 89100, Italy

## Background

The knowledge of the immune system role on melanoma has accelerated the translation of key advancements into medical breakthroughs like ipilimumab, an anti-CTLA4 immunomodulating antibody. Ipilimumab works amazingly well only in a limited number of patients and its effects on T-cell subpopulations as well as on immune response remains to be elucidated. Recently, it was described a new subset of immunomodulating T-cells, known as Double-negative T-cells (DNTs) expressing either αβ or γδ T-cell receptors (TCR) but lacking CD4,CD8,CD56. The DNTs contribute specifically to anti-tumor immunity since involved in immune regulation and tolerance acting as regulatory T-cells (Treg) and/or cytotoxic T-cells and they contribute to *in vivo* anti-melanoma immunity as previously reported [1-5]. However no data are available on their frequency in melanoma, as well as the effects of ipilimimumab on DNTs functional attitude in immunomodulation and on modulating their expression during the therapy. We aimed to evaluate the modulation of DNT frequency in Metastatic Melanoma (MM) patients treated with ipilimumab during the therapy in order to explore their potential role on clinical outcome and therapy response.

## Patients and methods

We carried out flow cytometric studies and statistical analyses on data of frequency of DNTs from 136 individuals, which included 16 healthy donors,30 MM patients who received ipilimumab as second line therapy, and 90 lymphoma patients. To evaluate the modulation of DNT during ipilimumab therapy we collected peripheral blood of MM subset at three time points: 1. Before start of therapy, 2. Before the 3th ipilimumab infusion, 3. After 2 months from the end of therapy. All patients provided their informed consent in accordance with the Declaration of Helsinki.

## Results

**Fig 1:**
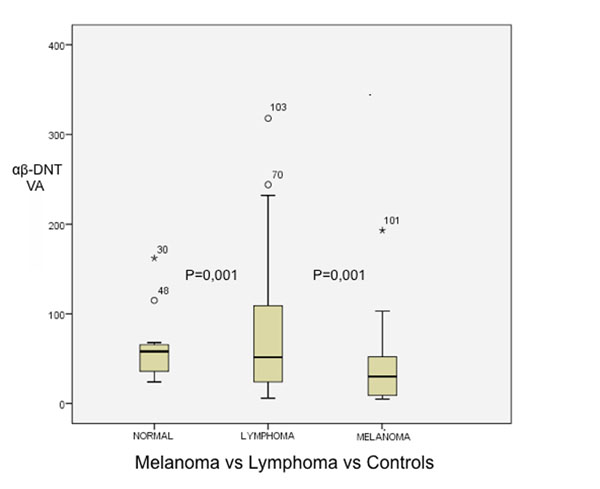
Circulating αβ-DNT frequency in Melanoma patients as compared with healthy controls and Lymphoma patients P=0,048

**Fig 2:**
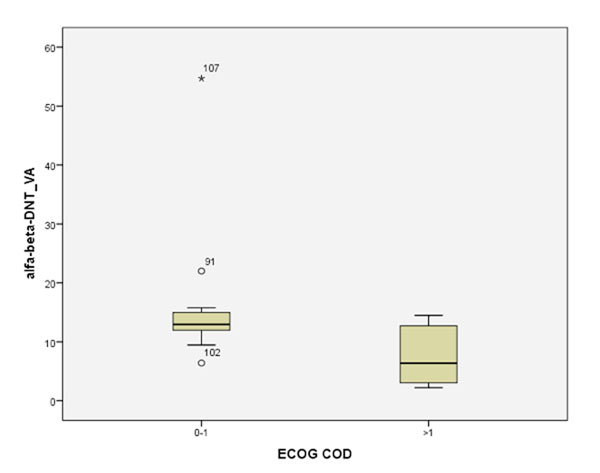
Circulating αβ-DNT frequency in Melanoma patients based of ECOG performance status.

**Fig 3:**
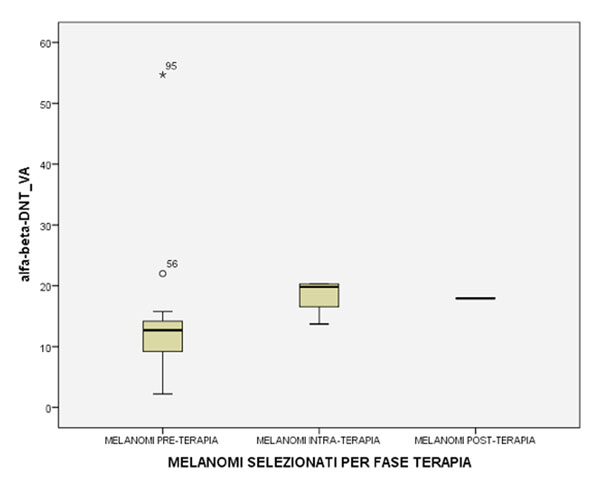
Circulating αβ-DNT frequency in Melanoma patients during the Ipilimumab treatment

We observed a significant decrease (p =0.001) of circulating αβ-DNT frequency in MM (13.49cells/ul±5.4) as compared with healthy controls (31.3cells/ul ± 3.4) and more interestingly when compared with Lymphoma patients (p=0.001) (fig.1). Furthermore, αβ-DNTs was significantly increased (p=0.048) in MM patients with ECOG performance status ≤1 as compared with >1 (fig.2). Finally, despite a low cluster was collected until the last time point sample, we observed a trend of significant increasing (p=0.083) in αβ-DNTs during the ipilimumab treatment (fig.3).

## Conclusions

This is the first report on evaluation of αβ-DNTs in MM patients and their modulation by an immunomodulating drug such as ipilimumab. Our preliminary data suggested a lower frequency of αβ-DNTs and a worse immunological impairment in MM compared to healthy and lymphoma subject as well as a trend of increasing of this T-cell population during the therapy. These results, supported by future studies, could estabilish the role of DNTs in MM patients and the significance of their increase during ipilimumab therapy.
